# Gendered Experiences of Parent–Child Communication on Sexual and Reproductive Health Issues: A Qualitative Study Employing Community-Based Participatory Methods among Primary Caregivers and Community Stakeholders in Rural South-Western Uganda

**DOI:** 10.3390/ijerph19095052

**Published:** 2022-04-21

**Authors:** Dorcus Achen, Viola N. Nyakato, Cecilia Akatukwasa, Elizabeth Kemigisha, Wendo Mlahagwa, Ruth Kaziga, Gad Ndaruhutse Ruzaaza, Godfrey Z. Rukundo, Kristien Michielsen, Stella Neema, Gily Coene

**Affiliations:** 1 Centre of Expertise on Gender, Diversity and Intersectionality, Vrije Universiteit Brussels, 1090 Brussels, Belgium; gily.coene@vub.be; 2 Faculty of Interdisciplinary Studies, Mbarara University of Science and Technology, Mbarara P.O. Box 1410, Uganda; vnyakato@must.ac.ug (V.N.N.); ekemigisha@must.ac.ug (E.K.); wolema@must.ac.ug (W.M.); 3 International Center for Reproductive Health, Department of Public Health and Primary Care, Faculty of Medicine and Health Sciences, Ghent University, 9000 Ghent, Belgium; cakatukwasa@gmail.com (C.A.); ruth@must.ac.ug (R.K.); kristien.michielsen@ugent.be (K.M.); 4 Department of Community Health, Faculty of Medicine, Mbarara University of Science and Technology, Mbarara P.O. Box 1410, Uganda; gruzaaza@must.ac.ug; 5 Department of Psychiatry, Faculty of Medicine, Mbarara University of Science and Technology, Mbarara P.O. Box 1410, Uganda; grukundo@must.ac.ug; 6 College of Humanities, Makerere University, Kampala P.O. Box 7062, Uganda; sheisim@yahoo.com

**Keywords:** parent–child communication, sexual and reproductive health, culture, gender

## Abstract

Open and positive parent–child communication about sexual and reproductive health (SRH) is known to reduce negative SRH outcomes for young people. However, socio-cultural influences can inhibit meaningful SRH communication. Restrictive gender norms threaten the SRH of adolescents, as they make adolescent boys more likely to engage in risky sexual behavior and make girls more vulnerable to negative SRH outcomes. This study intended to critically understand the impact of gender norms and expectations on parent–child SRH communication in rural south-western Uganda. Methods: The study adopted a community-based participatory approach using community stakeholder engagement meetings (n = 2), in-depth interviews (n = 12), and three focus group discussions with parents (n = 18). The study considered biological parents, step-parents, grandparents, uncles and aunties, as long as they were primary caregivers of adolescents aged 10–14. Results: Participants elaborated on the socio-cultural aspects that shaped their experiences of parent–child SRH communication such as cultural gender norms, religion, and media influences. They also referred to socio-economic challenges, lack of knowledge, and the role of peers and schools. Conclusions: There is need for community-based interventions to improve parent–child SRH communication to address the deeply rooted cultural and gender contexts in rural south-western Uganda.

## 1. Introduction

Gender socialization is a process that begins early in life and continues throughout the life course [1]. Gender norms play a crucial role in determining adolescents’ sexual and reproductive health outcomes [2]. Restrictive gender norms make adolescent boys more likely to engage in risky sexual behavior and make girls more vulnerable to negative sexual and reproductive health (SRH) outcomes [3]. In a study on gender norms associated with adolescent sexual behaviors in Uganda, both female and male adolescent participants agreed that men need sex more than women, husbands should be outraged if their wives ask to use condoms, that a man must have sex with other women even if things with his wife are fine, and that it was acceptable for husbands to beat their wives sometimes [4]. In Uganda, according to the UDHS 2016, 25% of adolescent girls aged 15–19 had begun childbearing, with higher rates in rural than in urban areas. Furthermore, men were reported to have more sexual partners than women, only 2% of women aged 15–49 reported that they had two or more sexual partners, compared to 21% of men in the same age group. More than one in five women aged 15–49 reported that they had experienced sexual violence at some point in time, compared with 8% of men [5]. The prevalence of HIV among individuals aged 15–64 in Uganda is 6.2%: 7.6% among females and 4.7% among males [5].

Open, positive communication between parents and adolescents on SRH issues is known to have positive effects on adolescents, their families, and society [6]. Research suggests that adolescents who discuss sex with their parents are more likely to make healthy decisions than those who do not talk to their parents very often [7]. A study that reviewed research on parent–child communication about sexuality and HIV/AIDS in sub-Saharan Africa indicates that women who participate in interventions to improve parent–child sexuality communication report significantly more frequent communication about sexuality with children than women in the control group. The intervention participants (parents and young people) reported that, as a result of their participation, the content of the discussions became much more specific and concrete about risk reduction strategies [8].

In rural south-western Uganda, as is the case in most parts of Uganda, traditionally it was very common for parents not to talk directly to their children about sex, but instead they let the paternal aunties and uncles impart such information to them [9]. SRH information for girls was usually passed on by their *Shwenkazi* (paternal auntie), and for boys by their *Shwento* (paternal uncle). In this process, emphasis was put on adhering to specific gender roles and expectations. These expectations prepared girls for a life of servitude in their marriages, where they were taught to be submissive towards husbands, while boys were left to explore and expected to tend to the domestic animals, play sports, and the like [10]. Research, however, suggests that, over time, and due to socio-economic changes in the extended family system, the “paternal auntie and uncle” institution has weakened as a primary medium for communication on sexuality [9]. The cash economy, formal education, family separation, and high levels of mobility have rendered it inactive [11]. This has created a gap in the way young people acquire sexual knowledge. Yet, with the emergence of HIV/AIDS and the rise of new media, informed and reliable sources of SRH information are crucial in ensuring that young people grow up in a safe and healthy environment [7,10].

In Uganda, the term Comprehensive Sexuality Education (CSE) tends to be associated with the promotion of relationships and sexual practices that are against the abstinence-only campaign. For example, following a media report in 2016, religious leaders in the country called for a ban on all aspects of school-based sexuality education [12]. This has delayed implementation of the Sexuality Education Framework that was developed based on extensive consultation with stakeholders that included religious leaders [12]. This paper describes the experiences of parents with SRH communication in rural south-western Uganda, and how they are shaped by gender norms and expectations.

## 2. Materials and Methods

### 2.1. Study Design

This study is part of the formative research for a project aimed at improving adolescent SRH through a participatory parent–child communication intervention in Uganda. The research is part of a collaborative project involving Ghent University (Belgium), Vrije Universiteit Brussel (Belgium), Mbarara University of Science and Technology (Uganda), and Makerere University (Uganda). The main aim of the project is to instill changes at the community and institutional levels to obtain better young adolescent SRH outcomes in Uganda. One of the main objectives is to improve communication between parents and young adolescents on SRH. For this study, we used a qualitative research design that employed community-based participatory research approaches (CBPR). This approach ensures community participation, community involvement, community consultation, and collaborative partnerships. As well, the community has to be engaged in the planning process, they have to be committed and there has to be inclusiveness in all activities. The goal of CBPR is to ensure that the research is relevant, culturally and practically acceptable, and mutually beneficial and that the voices especially of the vulnerable groups in the community are amplified.

### 2.2. Study Setting

The study was conducted among primary caregivers and community stakeholders in rural areas in south-west Uganda (Rwebishekye parish, Mbarara district). The parish has six villages (Muko, Rwebishekye I, Rwebishekye II, Kaburaishokye, Kikoma, and Mishenyi), and data were collected in all six. Rwebisheye parish is a close-knit rural community with limited basic resources and, in particular, limited SRH services. It has two government-aided schools, one primary and one secondary school. The parish has a minimal number of privately owned schools, whose access is limited, as they charge high tuition fees.

The data were collected between July and September 2020, in the initial stages of the COVID-19 pandemic in Uganda. The Government of Uganda had put in place restrictions in March to contain the spread of the virus; schools, workplaces, and shops had been closed, movement had been restricted, and only essential services had been in operation. This was important for the study because the pandemic had provided an opportunity for parents to stay with their children for an unprecedented amount of time. Because of this, they had experienced the benefits and challenges of parenting adolescents in ways that they would not have done if the children had been away at school.

### 2.3. Study Population

The main participants in this study were 19 female and 18 male primary caregivers who had adolescent children aged 10–14 years. One female parent and one male parent were selected from each of the six villages. The term “parent” in this community referred to anyone who took on the parental role in the lives of these adolescents; therefore, primary caregivers were considered. Consideration in the selection was given to biological parents, grandparents, step-parents, uncles and aunties. For each of the categories, a mother figure and a father figure were chosen for the in-depth interviews (IDIs) and focus group discussions (FGDs). The same categorization was used when selecting very young adolescents. A male and a female were each chosen from the above categories used for the parents.

The other group of participants considered were 15 community stakeholders; these included 9 male stakeholders and 6 female stakeholders. They were selected because they were people with influence and had a voice in the community. Two categories of leadership were considered: community leadership and technical leadership. Community leaders included councilors (n = 3), chairpersons (n = 2), village health teams (n = 3), and religious leaders (n = 3). Technical leadership included a health inspector (n = 1), a community development officer (n = 1), and teachers (n = 2).

### 2.4. Sampling

Purposive sampling was used in the identification and selection of information-rich cases. This involved identifying and selecting individuals or groups of individuals who were primary caregivers of adolescents aged 10–14 years and had some experience with parent–child SRH communication. Additionally, the study considered participants who were available and willing to participate, and were able to reflect and communicate experiences and opinions clearly [13]. Caregivers of adolescents were chosen based on their personal experience with parent–child SRH communication.

The community leaders were selected because they were opinion leaders in the community and responsible for the SRH of young people in the community [14].

### 2.5. Data Collection Methods

The study employed community-based participatory approaches. We held a community entry meeting at the beginning with community stakeholders to facilitate community buy-in for the project. This was particularly important since the subject of sexuality is sensitive and controversial. Therefore, it was essential to clearly explain the objectives and approach of the project. We worked with the Women’s Council Representative at the county level as the community contact person before and during data collection. Further, we worked with Village Health Teams (VHTs), who were already established in the community-level health structures and took on the role of community mobilizers.

*Community stakeholder engagement meetings*: First, we organized community stakeholder engagement meetings. In the first meeting, study evidence from the quantitative baseline survey was shared prior to the qualitative data collection. The baseline was a cross-sectional household survey carried out among 218 parent–young adolescent dyads. The survey explored how comfortable parents were discussing 10 SRH topics with their young adolescents, and whether they had already had such discussions with their children on these topics. Eight of these topics were presented at the stakeholder engagement meeting, as the analysis was still ongoing, and the results of the other two topics were still pending. The eight topics that were discussed were: general health and body hygiene, menstruation, wet dreams, romantic relationships, sexual pressure, pregnancy and birth control, HIV and other sexually transmitted infections, and sexual violence and reporting. These results were discussed in the form of a data party. A data party is a tool that allows those present to make meaning out of data collected on an issue. The survey presented statistics on whether they had discussed SRH topics with their children and how comfortable they were, and the data party allowed us to make meaning of the statistics from the experiences of the community.

In a second stakeholder meeting, an appreciative inquiry session was held. Appreciative inquiry is a developmental tool used as a process to develop positive change in communities. This tool encourages groups to ask, learn, and build on their strengths as communities, rather than to emphasize problems [15]. Stakeholders were invited to talk about the parenting practices that the community was proud of. They were asked to talk about moments in their lives as primary caregivers that had made them feel like good parents. In this meeting, a third session was held in which a social network analysis was undertaken. Stakeholders in the meeting were tasked to draw and talk about their children’s social networks [16].

Finally, IDIs and FGDs with primary caregivers were completed. We conducted 12 IDIs with father figures (n = 6) and mother figures (n = 6). In addition, three FGDs were carried out with primary caregivers: one with mother figures (n = 6), one with father figures (n = 6), and one mixed (n = 13).

### 2.6. Study Tools

Interview guides and meeting guides were used to collect data. The meeting guide with community stakeholders focused on a data party to disseminate results from a quantitative survey as part of the larger study. Appreciative inquiry was used to bring to light the parenting practices that the community was proud of, and a social network analysis to understand if parents knew their children’s social networks. The parent guides focused on practices and experiences of parent–child SRH communication.

### 2.7. Data Analysis

All the data collection tools were translated into the local language (Runyankore-Rukiga), and all interviews were recorded and carried out in the local language by research assistants and the first author. Interviews were then transcribed and translated into English. An initial coding frame was then developed. The first step was to manually read through interview guides and a few interview transcripts, from which initial themes were developed. The rest of the transcripts were then read to expand the codes. The developed themes were then entered as codes into NVIVO software. Codes were derived inductively from the data and deductively from interview tools. The data were then examined for differences and similarities. Patterns and categories were then merged into some main themes such as methods of SRH communication, gender roles, religion, culture, financial challenges, and lack of knowledge.

### 2.8. Study Limitation

This being a qualitative study, there could be bias attributed to social desirability during the focus group discussions where there may be over-reporting of desirable attributes or under-reporting of undesired attributes within a group. Confirmation bias can be inherent to any quantitative study where people may ignore new information that contradicts existing beliefs. Extreme response bias may also be present, especially in the setting of strong gender and cultural beliefs. Furthermore, as questions and answers were translated from the native language to English, misinterpretations could be present; to reduce this, we ensured interviewers were competent in the local language spoken in the area.

## 3. Results

Table 1 illustrates the socio-demographic characteristics of the primary caregivers who participated in the study. The gender split was almost even (51% male and 49% female). Their ages ranged from 28 to 80 years. Most of the respondents were aged 31–40 or 51–60. The respondents were a reflection of the highly religious nature of this community, which is predominantly Anglican. Two-thirds (67%) identified as Anglican, 19% as Catholic, 11% as Pentecostal, and 3% as Muslim. The marital status of the respondents was such that 78% were married, 13% were widowed, and 11% were separated. Around 84% of the respondents were peasant farmers, while 16% had other occupations. About 41% had not received any form of education, 43% had acquired some form of primary education, 11% had acquired some form of secondary education, and 5% had attained tertiary education.

All parents were primary caregivers of adolescents aged 10–14. Nearly half (46%) of them were biological parents, of which 59% were from two-parent homes, while 41% were single parents. A further 19% were grandparents, 19% were step-parents, and 6% were either an auntie or uncle. There were not very many biological parents because some young parents have left their children in the village to go and look for work in the cities and bigger towns.

### 3.1. Parents’ Experiences of Parent–Child Communication on SRH Issues

In this section, the findings are presented to show the gendered experiences of parents with parent–child SRH communication. They highlight their experiences and the impact of gender roles, norms, and expectations that society places on girls and boys in the various spheres of society in rural south-western Uganda. The themes are organized first to show the methods that parents use to communicate with their children, and then more critically delve into the influence of various aspects of parent–child SRH communication; it looks at gender and cultural norms, religion and media, work and financial challenges, the lack of knowledge, and the role of schools and peers.

### 3.2. Experiences of Parent–Child SRH Communication

In the first theme we talk about how parents communicate with their children about SRH issues in this rural south-western Ugandan community. Parents revealed that they communicate about SRH issues with their children using threats, intimidation, and quarreling, spanking, and scare tactics. Traditionally among Ankore (a dominant ethnic group in the region) it was demanded that children fear and respect parents and that parents show they have a hold on their home. The parents in the interviews mentioned that when they instilled fear in their children, the children became afraid of the consequences of negative SRH decisions such as pregnancy. It was believed that this fear deterred them from engaging in such activities:

*“… for instance, you tell the child if I find you playing around with boys, I won’t pay your school fees for next term, and then she will stop because then she will fear to do wrong.”*

(Father, IDI)

Additionally, parents also mentioned that they monitored and policed their children’s activities. They were constantly looking out for who the friends of their children were and the kind of groups they engaged with. They also monitored the places the children went, and how they spent their time. Traditionally, how a child behaved in public was an extension of how their parents raised them. This notion was used to tailor the kind of SRH communication they had with the child.

*“Monitor your child, know their groups, and limit them in case you notice bad groups. You might find that they have joined bad groups and are now betting, drinking alcohol, and going around with the opposite sex, and it is only through monitoring that you can know what they are involved in and what to talk to them about.”*

(VHT, stakeholder meeting)

*“When you see that they have started spending time with the opposite sex, walking at night, and joining bad groups, you have to warn them. I told my daughter that if she gets pregnant from those bad groups of hers, she shouldn’t come back to my house, since she won’t be a child anymore.”*

(Father, IDI)

The communication was triggered by events. The parents did not talk with their children about SRH issues until they encountered an event that forced them to. For instance, if they saw their sons hanging around with girls.

*“The neighbor said he saw my son with a girl, and so I knew I had to talk to him. I told him that if he makes anybody’s daughter pregnant, he is on his own. I was so angry and quarreled at him.”*

(Mother, FGD)

The parents mentioned that this era of HIV has forced them to have to talk about sex to their children, because boys playing around with girls could mean they get HIV, and they will be sick forever. HIV/AIDS has facilitated communication with children and broadened the scope from prevention of pregnancy to prevention of sexually transmitted infections/HIV, which now also includes boys’ SRH issues.

*“You see now these days there is HIV/AIDS, and so we have to talk to our children and make sure girls don’t play with boys and boys don’t play with girls. We have to tell them to be careful because nowadays we have to worry about HIV/AIDS. Those days our only worry was pregnancy, and with pregnancy you produce the child and your life continues, but HIV/AIDS stays with you forever.”*

(Woman councilor, stakeholder meeting)

### 3.3. Gender Roles and Cultural Norms with Regard to SRH Communication

Under this theme we talked about the role of gender norms in parent–child SRH communication, what the specific roles, norms, and expectations are for girls and boys, for mothers and fathers, how culture reinforces these, and their impact on parents’ discussion of SRH issues with their children. Parents mentioned that keeping the children busy at home with chores facilitated parent–child communication. A good girl was one who knew her way around house chores and knew how to keep the home and care for her siblings. A good boy knew how to graze cattle. The roles of girls were restrictive and were meant to keep them around the house where their parents could watch them, as well as to prepare them to be good wives. The parents said it also gave the parents time with their children to teach them specific gender roles, and they considered this a type of parent–child SRH communication.

*“You have to make sure your child is busy at home with chores so that they don’t loiter and also so that they can do chores with their parents, and this helps parents to talk to their children about being responsible.”*

(Stepmother, FGD)

*“The girl must be close to her mother to learn female chores—sweeping and cooking, for instance—and this will help her learn from her mother how to be a good woman and wife. It also gives her mother the chance to talk to her about girls’ issues.”*

(Chairman, stakeholder meeting)

Parents also mentioned that the reason why fathers did not talk to their daughters and why they did not know about girls’ issues was cultural. Mothers, aunties, and senior women had talked secretly to girls about their SRH issues, and no boys or men were allowed when this happened. Men grew up to be fathers who did not know anything about “girls’ issues” and thought it was not their place to talk about them. As such, they did not know what to say or how to talk about SRH issues with their daughters.

*“In our culture, fathers didn’t talk to their daughters about these issues. Men and boys were never present when they were talking to girls about menstruation, sex, and all these other issues. It was not allowed, and so that is why I don’t talk to my daughter. I leave her issues for the mother.”*

(Chairman, stakeholder meeting)

While participants agreed that culture did not give men any opportunity to learn and talk about girls’ issues and women to learn about men’s sexual issues, they argued that things were changing, and in the event of single-parent homes, the parent—whether only a mother of boys, a father of daughters, or a single parent of both—should still be able to talk to all their children about SRH issues equally.

*“…while I agree that because of culture men could not talk to their daughters, things are changing, and these days there are single-parent homes, and so if you are a single father with only daughters, will you allow your daughters to stray because of culture? No, you have to find a way of talking to them.”*

(Teacher, stakeholder meeting)

*“I have both daughters and sons, but their mother is very tough, and they are freer around me and can talk to me about anything, and so because of that I am the one who talks to them, I even buy them pads. Things are changing.”*

(Father, IDI)

Participants mentioned that culture was fading, and that was one of the major challenges of parent–child sexuality communication. Traditionally, there was a system where when children had started to become adolescents, their parents sent them away to their paternal aunties (for girls) and paternal uncles (for boys) to go and talk about becoming a woman and becoming a man, what they must do, and how they must conduct themselves. However, now even these paternal aunties do not know what to do, and some of them are not even conversant with the traditional culture.

In addition to the above, values such as virginity were taken seriously in traditional Ankore. A girl who was a virgin when she got married brought great pride to the family, and it was a sign that her parents had raised her well; one who was not a virgin brought shame and disgrace to the family. In today’s society, no one cares about virginity, and young people do not listen to their parents and do as they please.

*“In the traditional society, the paternal aunty and uncle were obliged to talk to and groom children about sexual and reproductive issues, and so when parents thought children were ready, they would send them to their aunties/uncles. Parents did not really talk to their children about sexuality issues, and this is the system in which the parents were raised, and so it makes it difficult for some of us to talk to our children.”*

(Father, FGD)

*“As well, virginity was paramount, at first intercourse, then after a girl got married, her family would be given a goat, and this was a source of pride. If she was not, a spear would be put around the bedsheet and brought out for everyone to see, and the girl’s family would be put to shame. And so this ensured parents to preach abstinence and to ensure that their daughters did not have sex before marriage, but today’s society does not value virginity, and children do not listen, and so parents don’t know how or what to talk about.”*

(Aunty, IDI)

*“You see those traditional people put more emphasis on a girl’s virginity, and so parents would get a lot of dowry in the form of cows, and so they would make sure that their girl child grows very well without losing her virginity so they would get more cows. But now things have changed and it’s not important and we cannot warn girls, and even though we try, they don’t listen to us at times.”*

(Grandfather, IDI)

Traditionally, society was harsher on girls for the negative consequences of SRH such as pregnancy. It meant she had brought shame on her parents, whereas for boys, it meant that he had proved that he was a man. In the findings, some of the parents emphasized the importance of talking to girls and ignored boys because, apparently, boys did not really have challenges, even though it was clear in some interviews that boys also had challenges.

*“Boys don’t have challenges. They make girls pregnant, but that is not really a challenge because boys do not carry the pregnancy, they do not drop out of school because of pregnancy, and it is a sign of manhood. For girls, however, it means shame, broken virginity, her value has reduced, and so it is important to talk to girls.”*

(LC1 chairman, stakeholder meeting)

*“Boys making girls pregnant is not a bad thing because children are a blessing and it shows that he is a man. So when it happens, you get mad at him, but it is not the end of the world. As for the girl, she will get pregnant, and the community will see, and she won’t be able to get a good home, and she will shame the parents, so it’s important to talk to girls more.”*

(Father, FGD)

Some participants disagreed with the view that boys do not have challenges, since they are more prone to other risks such as betting, alcoholism, and drug abuse. Moreover, when they make a girl pregnant, they “spoil” their name and have to become a provider early in life.

*“Boys also have challenges, they are the ones whom you find betting their school fees, abusing drugs, and drunk from alcohol.”*

(Mother, FGD)

*“You see because of poverty, some boys have been forced to drop out of school and work so that they too can provide for the family.”*

(Teacher, FGD)

Some of the parents argued that it was important to talk to both boys and girls equally because the boys spend time betting, taking alcohol and drugs, and watching bad movies in the trading center.

*“…our boys spend a lot of time in the trading center, which is very close and congested. They spend it betting with money which I don’t even know where they get it from. It encourages them to be thieves. And then alcohol is another problem in the trading center, and so if we don’t talk to them, they will all get spoilt.”*

(Grandmother, IDI)

*“…there are even those places in the trading center where they watch bad movies which are not for their age and then they want to go and try the things they have been watching on the girls in the village.”*

(Father, IDI)

### 3.4. Religion and Media

Religion was an important aspect of the community. It plays a major role in the socialization of children. Every parent belongs to a religious community and attended church regularly and took religion as an integral part of every part of their lives. The church emphasized abstinence from Bible teachings. The church leaders also talked about avoiding pregnancy and HIV, so parents mentioned that this made it easier for them to talk about, it since the children had already heard about it from church. Religious doctrines were to be followed unconditionally:

*“Church has really helped us. In Sunday school they tell girls to avoid boys, and they tell boys to avoid girls. They also tell them to avoid pregnancy and HIV, and when they have heard these things from church it is like the word from God, and then me as a parent I am just adding on what the church has said.”*

(Auntie, IDI)

*“We also have mothers’ union and fathers’ union at church, and they talk to us parents about how to be good parents and how to raise well-behaved children in the community. In some of our meetings, they talk about teaching our children to abstain from sex and to be clean. Even when they see that you as a parent are not behaving well, they talk to you and you change and become a good example for your children.”*

(Stepfather, FGD)

*“Also when they make girls pregnant, even if they continue to study, your name is stained and your family is stained and it will affect him in the future when he wants to get married in church.”*

(Teacher, stakeholder meeting)

In addition, media are mentioned as a newer culture that parents suggested was invading the community. It exposed their children to the outside world and made it difficult for parents to protect their children from it.

In the findings, parents mentioned that media sources such as television and phones had made it difficult for parents to talk to their children about SRH issues. This is because after they had heard and watched something on television and on the phone, they did not listen to what their parents told them. Parents said that children trusted what they watched, saw, and heard from the media more than what their parents told them, so parents found that the media were a problem in that respect.

*“When they see things on TV or from the phone, they start feeling that they know everything and will not listen to what you are telling them.”*

(Mother, FGD)

*“They also watch pornography on the internet, and if in that pornography there is an old man having sex with a young girl, there is no way you will convince her that it is wrong for a young girl to have sex with big men.”*

(Father, IDI)

The study also explored other experiences apart from cultural experiences. The participants also talked about how personal issues and the community had influenced their experiences with parent–child sexuality communication

### 3.5. Work and Financial Challenges

Parents also mentioned that they were often too busy with work and were unable to make time to sit down and talk to their children about issues of sexuality even though they would have liked to. Usually when they come back from work, it is very late, the children are already asleep, and the parents are tired. Children also spend too much time at school and only get very short holidays, making it even harder for parents to create time.

*“Establishing the relationship between a child and a parent in talking about sexuality issues might become difficult because in most cases children don’t stay at home and when they come back for holidays, they find that their parents are also very busy with work and have no time for the children.”*

(Father, IDI)

*“We honestly don’t have time, even if we wanted to talk to the children, you get so busy during the day and when you want to create time, it is time for bed or they are at school.”*

(Mother, FGD)

To counter this, some parents argued that if you really wanted to, you could find time to talk to your children. They said that parents were mostly afraid of talking to their children and used having no time as an excuse. They mentioned, for instance, that parents could create time at weekends when they could plan to do chores together before the parents go to work or even Sundays after church.

*“I think some parents just fear to talk to their children and they use the excuse of they don’t have time because if you really wanted to talk, they can make time. You can do chores together, especially the mothers, even after church on Sunday, you can talk.”*

(Auntie, IDI)

Respondents also mentioned financial challenges as a major influence on parent–child sexuality communication. Some parents said they did not talk about certain topics because if they started talking to them, they had to follow up and buy their children the needs attached to that SRH issue, but they could not afford it. They said this was especially for girls because as they grow, they start to take how they look seriously and want to keep up with society’s beauty standards, which are expensive for parents. The parents also worry because if they cannot afford this, it makes their daughters try to get these things from older men, which leads them to having sex early.

*“Money is a challenge, and that is why sometimes even if we want to talk about certain issues, we cannot. Because how do you talk to children about menstruation when you cannot buy pads? Because after you have talked about it, you have to buy them, and if you do not have the money, you will be embarrassed.”*

(Pastor, stakeholder meeting)

*“Parents can’t afford to provide all the basic needs like pads, clothes… you know most of the girls want to look smart and because we can’t afford, they start to admire a lot of things hence start to look for men who will provide for them these things.”*

(Uncle, IDI)

*“You see us when we were young, our parents taught us to keep a cloth as a pad during our periods. We had to keep the cloth clean. But these days even if you tell them about the cloth they will not allow because their friends at school are using pads, and so it makes it difficult for us to talk about these issues.”*

(Mother, IDI)

*“…the problem is because we cannot afford to buy for them the things that they want, they go and look for them from sugar daddies, and these men end up asking for sex, and the girls have no choice. And as the parent you feel somehow that you have failed…”*

(Father, IDI)

*“But the problem is we do not have enough money to help our own children. If you have a tank within your homestead, then your girl-child does not have to go far to fetch water. But now she has to go out with a jerry can to fetch from a far off distance and yet there are these boys who are ready to disturb the child. The boy will tell his friend, ‘That person’s daughter fetches water from this well. If you give her just 2000 shillings, you will be able to sleep with her.’ That is how the youth are nowadays.”*

(Auntie, FGD)

### 3.6. Lack of Knowledge, and the Role of Schools and Peers

Parents admitted a lack of knowledge on SRH issues. Mothers said they did not know much about boys’ issues, and fathers said the same about girl’s issues. Thus, it made it hard to talk about these issues, because when they were growing up in Ankore, the aunties only talked to the girls in private. Therefore, boys never got to learn about women’s issues, and girls never got to learn about boys’ issues. Further, the parents said that most of them had not really had any education, so they did not get the chance to attain the knowledge at school either. Parents, therefore, found it difficult to confidently talk about issues they did not know much about. They also had the idea that children often knew more than their parents.

*“I can’t be able to talk about what I don’t know.”*

(Grandmother, IDI)

*“As her father, I don’t know about menstruation and pads and things like that, so how am I supposed to talk to my daughter about these things? …And this is worse in homes where a man lives with only daughters.”*

(Father, IDI)

*“These children of ours know more than us from school, so how do you even start talking about issues you don’t know very well with someone who has even learned them from school? Some of us did not get the chance to go far with school, and you know in our culture talking about sex is almost a taboo, especially between patents and their children.”*

(Grandmother, FGD)

The parents believed that their individual behavior had an impact on parent–child sexuality communication. They thought that if the parent behaved responsibly around children, then the children would try to be responsible too. However, if they behaved irresponsibly and practiced gender-based violence and alcoholism, the children would try to be like that too because children copy and learn from the actions of their parents. In that regard, communication was much more than just talking.

*“There is a man here in this village who goes to the bar with his son, and the son watches drunk people being inappropriate in the bar, and he watches his father too. So how will the father who is supposed to be the head of the household then correct him and talk to him when the child is learning from the father.”*

(VHT, stakeholder meeting)

*“If your child sees you beating the mother every day, he will start to think that it is normal, and after that it doesn’t even matter if you tell him otherwise. And children are always paying attention.”*

(Chairman, stakeholder meeting)

To add to this issue, parents mentioned that some parents had really small houses in which they lived with their children, so the parents did not have enough privacy to be intimate. Because of this, some children got to see their parents having sex and, therefore, copied with their friends of the opposite sex what they had seen from their parents.

*“You see some of us have very small houses. You live in a one-bedroom house with your husband and all the children and at night when your husband wants to have sex, you cannot say no, and you think the children are sleeping, but they can hear. And during the day when you are away, they will go and try those things with their friends and cause problems.”*

(VHT, stakeholder meeting)

Parents mentioned that in school their children were taught about adolescence and issues that boys and girls would face. They taught them about keeping clean and menstruation. In schools, there was also the Presidential Initiative on AIDS Strategy to Youth (PIASCY), designed to prevent the spread of HIV/AIDS and to mitigate its impact on primary and post-primary educational institutions in Uganda. The parents said this was very helpful in teaching their children about issues that parents did not know about, so the parents could concentrate on the issues at “home.” This was important because the parents would just complement some issues and would also be confident that if there was an issue they did not know about, it had been talked about in school.

*“Children are always at school. At school, they teach our children about adolescence and things like menstruation and other things which we don’t know about because we did not go to school, and then us we can talk to them about things that we know here at home.”*

(Mother, IDI)

*“There is also PIASCY at school which teaches our children about those sexual and reproductive health issues, and now some of them even know more than us. So we focus on teaching them the things that were taught to us about growing up in Ankore like cooking, cleaning, and grazing cows.”*

(Mother, IDI)

Parents mentioned that peer groups also had an impact on parent–child sexuality communication. This is because, during adolescence, children are closer and listen to their friends more than they listen to their parents; irrespective of what parents talk to them about, they listen to their friends. Therefore, when a child is involved with bad peer groups, it makes it difficult for parents to talk to them about SRH issues.

*“Adolescents form peer groups, and when they are in these groups they learn bad manners. They start having sex together, betting, alcohol, drugs, stealing, and all kind of bad manners in this village, and so even when we try to talk to them, they only listen to their friends.”*

(Mother, IDI)

*“… another challenge with peer relationships is that boys have always been pulled to video halls, watching football matches together as peers and from these they learn a lot of things that may be of a negative impact in their life, especially these movies they watch on TVs because they always want to copy and put into practice whatever they watch and they end up falling prey!”*

(Uncle, FGD)

*“The women also no longer talk to their children. You can find that there is this child whose mother is in the bar, the woman does not even know where the child has spent the day. She does not know the time at which her daughter arrived at home because she was also in the bar, and yet the child is getting spoilt in peer groups. The women do not want to sit with their children and tell them about these peer groups and the dangers associated with them. That is no longer there.”*

(Grandfather, FGD)

While most parents agreed that peer groups were a menace, some mentioned that peer groups could be good and could complement parent–child sexuality communication. Peer groups where children prayed and studied together were seen as constructive in deterring children from negative SRH activities and reinforcing the teachings of parents. Therefore, good peer groups were seen as a protective factor.

*“Children can learn from peer groups about menstruation, hygiene and body changes, shaving pubic hair, know about sperms, and voice deepening, and some of these things when we come to talk to them, we find that they are already doing them because they learned from their friends.”*

(Mother, FGD)

*“Also friends who encourage their friends to pray, to dress well and who help each other study also usually prevent girls from being with boys because they listen to their parents.”*

(Mother, IDI)

The neighbors in the community also influenced parent–child sexuality communication, according to parents. In the community, children are often allowed to go to the neighborhood and play with friends and interact with the neighbors. However, parents say that adolescents more often than not learn bad manners from neighbors that counter what they have been taught at home.

*“You will raise your child in the best way you can, and they will still learn bad manners from the neighbors.”*

(Mother, IDI)

*“Also sometimes you do not know what the neighbor is teaching their children, or maybe while you labor to talk to your children, the neighbor does not even care, so when your child goes to interact with the neighbor, they learn bad manners and all your effort will be for nothing.”*

(Auntie, IDI)

## 4. Discussion

This was a qualitative study aimed at generating critical understanding of the experiences of parenting and the role of gender norms on parent–child SRH communication in rural south-western Uganda. The findings of the study highlight the influence of cultural gender norms, religion, media, socio-economic challenges, and a lack of knowledge, peers, and schools.

In this study, the nature of communication between parents and their adolescent children was found to be authoritative and event triggered. SRH communication is often compounded by threats, fear, scare tactics, and harsh warnings [17]. Traditionally, parents did not talk to their children; the role was assigned to paternal uncles and aunties [10]. However, due to socio-cultural changes and the emergence of HIV/AIDS and new media, parents have to find ways of communicating with their children. The perceived danger of HIV/AIDS and the media for adolescents’ SRH, combined with the rigid nature of the environment that local culture and religion have created, have led parents to be tough and instill fear in their children, as they do not know how else to communicate with them.

The study suggests that the bulk of parent–child sexuality communication in rural south-western Uganda is based on gender norming that is highly influenced by culture. Ifi Amdume argues in his exploratory essay that traditional cultures practice customs that regulate women’s sexuality and meddle with and fight over women’s bodies, and such is the essence of such restrictive gender roles, norms, and expectations placed on girls and women [18]. In a study of very young adolescents’ perceptions on growing up in rural south-western Uganda that explored the gender roles and expectations of very young adolescents, the findings showed that the activities ascribed to girls are protective and restrictive, such as doing household chores such as cooking and cleaning, which also prepares them to be good and submissive. On the other hand, boys are allowed more freedoms that allow them to explore, such as grazing animals or going to the trading center [11]. In another study on attitudes to gender equality, sexual behavior, positive sexual experiences, and communication about sex among sexually active and non-sexually active adolescents in Bolivia and Ecuador, adolescents who considered gender equality important reported increased use of contraceptives and positive sexual experiences, and mentioned that it was easier to talk about their SRH issues. It is important to note that these correlations remained consistent for both boys and girls [3]. These findings re-emphasize the important role that gender plays in the SRH of adolescents [18].

The community in which the study was carried out is a very religious community, and religious beliefs reinforce cultural beliefs that make it difficult to talk about SRH issues. The church emphasizes that sex and boys are a sin, and that virginity is to be kept until marriage. This behavior is mostly emphasized for girls. Similarly, Moore, in his research on beliefs about sex and parent–child–church communication among church-based African American youth talks about the church emphasizing that sex is a sin and that virginity is to be kept until marriage. While this practice might encourage adolescents to wait to engage in sex until later when they are married, if they do have sexual relations before marriage, it is difficult for them to have the conversation openly with their parents [19].

Parents could not afford to expose their children to electronic media, as the devices were expensive, so they could not afford them. Additionally, most of the parents were illiterate and did not know how to use them. As such, they concentrated on the negative aspects of the media and discouraged their usage. Parents mentioned that children often sneaked into the trading center to be able to access television and the internet, which in turn exposed them to sports betting, drug abuse, and pornography. Some studies on media and SRH in Africa assert that media shape and influence the behavior of adolescents. They observe, imitate, and learn sexual behavior in particular, and as such are likely to engage in risky sexual behavior, as they can do this unsupervised at home or at a café [20]. Other studies, however, have shown that while high levels of social networking usage could have negative implications for adolescents, social networking with parents potentially strengthens parent–child relationships, which has positive outcomes for adolescent SRH [18,19].

In the study, we saw that community leaders and stakeholders who were teachers, government leaders, and church leaders participated in their capacity as parents rather than leaders. They too were going through similar struggles as parents and were tied to the gendered norms and expectations of society. This makes it difficult for them to be able to change how parents talk to their children about SRH issues to bring about gender equality. In a study undertaken with teachers on teaching sexuality education in Ugandan schools, teachers were found to be uncomfortable teaching sexuality education, because some of the content conflicted with cultural values and beliefs, and as such the teachers reinforced the cultural values with which they were more comfortable [21].

The study suggests that most of the parent–child sexuality communication is undertaken by mothers. In Uganda, it is said that a child’s behavior is adopted from the mother; therefore, a child’s bad behavior is a display of their mother’s behavior [22]. In the event that the child—boy or girl—went astray, the community and even the father would blame the mother for neglecting her role. However, the mothers said they preferred talking to their daughters instead of to their sons because of a lack of knowledge of boys’ SRH issues. Traditionally, girls’ SRH issues and discussions were restricted to girls and women, and fathers were not allowed to talk to their daughters about these issues. Additionally, the boys sometimes did not take their mothers seriously because they were women, and this made the mothers afraid [11]. Now, with changes in the family structure to more nuclear families, and because some fathers are now single (divorced, separated, or widowed) fathers of daughters and sons, fathers are also making attempts to talk to their children, although they are limited by traditional norms, a lack of knowledge on sexuality issues, and the still very gendered norms and expectations present in the socialization of adolescents. Because of this, they do not know what to say, how to say it, or when.

The study findings bring to the fore the different interacting inequalities that make it difficult for parents to have positive and open SRH communication with their children and worsen gender inequality. Gender lies in social identities; in a patriarchal society, the main role of emphasizing gender roles, norms, and expectations is to reinforce power relations [23]. Thus, compounding factors such as a lack of knowledge, poverty, cultural barriers, religion, peers, limited exposure to technology, and gender-based violence work to broaden the gender inequality gap in parent–child SRH communication. This makes it hard to have open and positive conversations. These compounding factors strip parents, especially fathers, of their social identity as heads of households, providers, and responsible fathers, so they lose power and desperately reinforce gender roles, norms, and expectations in an ambiguous claim to power [24].

## 5. Conclusions

The findings from this study give an understanding (from the parents’ and community stakeholders’ perspectives) of the influence of gender on parent–child SRH communication. The socialization of girls and boys in rural south-western Uganda puts gender roles, norms, and expectations at center stage, and this has a major impact on how parents talk to their children about SRH issues. The findings echo the need for research and interventions that emphasize gender equality to improve parent–child SRH communication. Additionally, interventions need to be creative in engaging the community, because gender relations are trapped in various aspects of life, and members of the community have been socialized with these gendered values throughout their life course. As such, achieving gender equality in parent–child communication on SRH issues will not be easy.

Gender stereotypes can be addressed through community programs that engage boys in activities traditionally reserved for girls and girls in activities reserved for boys. Gender equality in parent–child SRH communication can also be enabled by targeting parents, for instance, through preaching in places of worship.

## Figures and Tables

**Table 1:** Socio-demographic characteristics.

Socio-Demographic Characteristics of Parents (37 Participants)	Categories	N (%)
**Age**	21–30	3 (8%)
	31–40	11 (30%)
	41–50	7 (19%)
	51–60	10 (27%)
	61–70	3 (8%)
	71–80	3 (8%)
**Sex**	Male	19 (51%)
	Female	18 (49%)
**Marital status**	Married	28 (78%)
	Widowed	5 (13%)
	Separated	4 (11%)
**Formal education level**	No formal education	15 (41%)
	Some primary education	16 (43%)
	Secondary education	4 (11%)
	Tertiary education	2 (5%)
**Religion**	Anglican	25 (67%)
	Catholic	7 (19%)
	Pentecostal	4 (11%)
	Muslim	1 (3%)
**Occupation**	Peasant farmer	31 (84%)
	Other	6 (16%)
**Parent type**	Biological parent	17 (46%)
	Grandparent	7 (19%)
	Step-parent	7 (19%)
	Uncle/auntie	6 (16%)
